# *catena*-Poly[[[penta­aqua­cerium(III)]-μ-pyridine-2,4,6-tricarboxyl­ato-κ^4^ *N*,*O* ^2^,*O* ^6^:*O* ^6′^] tetra­hydrate]

**DOI:** 10.1107/S1600536812015887

**Published:** 2012-04-18

**Authors:** Shahzad Sharif, Islam Ullah Khan, Samia Zaheer, Seik Weng Ng

**Affiliations:** a Materials Chemistry Laboratory, Department of Chemistry, GC University, Lahore, Pakistan; b Department of Chemistry, University of Malaya, 50603 Kuala Lumpur, Malaysia; c Chemistry Department, Faculty of Science, King Abdulaziz University, PO Box 80203 Jeddah, Saudi Arabia

## Abstract

The Ce^III^ atom in the title compound, {[Ce(C_8_H_2_NO_6_)(H_2_O)_5_]·4H_2_O}_*n*_, is *N*,*O*,*O*′-chelated by the carboxyl­ate trianion and is coordinated by five water mol­ecules; a carboxyl O atom from an adjacent trianion bridges the Ce^III^ atom, resulting in a chain running along the *a* axis. The nine atoms surrounding the metal atom comprise a tricapped trigonal-prismatic polyhedron. The coordinated and lattice water mol­ecules inter­act with each other and with the carboxyl O atoms by O—H⋯O hydrogen bonds, generating a three-dimensional network.

## Related literature

For the isotypic Sm^III^, Eu^III^, Tb^III^ and Ho^III^ analogs, see: Wang *et al.* (2007 ▶). For the synthesis of 2,4,6-pyridine­tricarb­oxy­lic acid, see: Syper *et al.* (1980 ▶). 
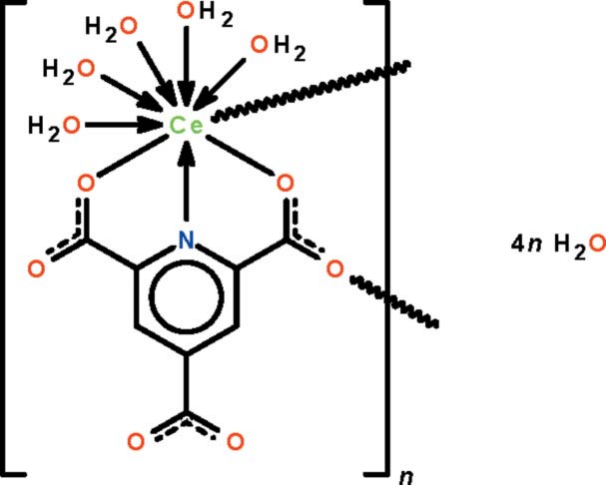


## Experimental

### 

#### Crystal data

[Ce(C_8_H_2_NO_6_)(H_2_O)_5_]·4H_2_O
*M* *_r_* = 510.37
Orthorhombic, 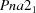

*a* = 6.8437 (3) Å
*b* = 13.3207 (5) Å
*c* = 17.9045 (7) Å
*V* = 1632.23 (11) Å^3^
*Z* = 4
Mo *K*α radiation
μ = 2.87 mm^−1^
*T* = 293 K
0.21 × 0.06 × 0.04 mm

#### Data collection

Bruker APEXII diffractometer
Absorption correction: multi-scan (*SADABS*; Sheldrick, 1996 ▶) *T*_min_ = 0.584, *T*_max_ = 0.894
19512 measured reflections
3690 independent reflections
3336 reflections with *I* > 2σ(*I*)
*R*_int_ = 0.034

#### Refinement

*R*[*F* ^2^ > 2σ(*F* ^2^)] = 0.033
*wR*(*F* ^2^) = 0.078
*S* = 1.07
3690 reflections
226 parameters
25 restraints
H-atom parameters constrained
Δρ_max_ = 0.68 e Å^−3^
Δρ_min_ = −0.51 e Å^−3^
Absolute structure: Flack (1983 ▶), 1757 Friedel pairs
Flack parameter: −0.05 (2)

### 

Data collection: *APEX2* (Bruker, 2011 ▶); cell refinement: *SAINT* (Bruker, 2011 ▶); data reduction: *SAINT*); program(s) used to solve structure: *SHELXS97* (Sheldrick, 2008 ▶); program(s) used to refine structure: *SHELXL97* (Sheldrick, 2008 ▶); molecular graphics: *X-SEED* (Barbour, 2001 ▶); software used to prepare material for publication: *publCIF* (Westrip, 2010 ▶).

## Supplementary Material

Crystal structure: contains datablock(s) global, I. DOI: 10.1107/S1600536812015887/xu5510sup1.cif

Structure factors: contains datablock(s) I. DOI: 10.1107/S1600536812015887/xu5510Isup2.hkl

Additional supplementary materials:  crystallographic information; 3D view; checkCIF report

## Figures and Tables

**Table 1:** Hydrogen-bond geometry (Å, °)

*D*—H⋯*A*	*D*—H	H⋯*A*	*D*⋯*A*	*D*—H⋯*A*
O1*w*—H12⋯O6*w*^i^	0.84	2.00	2.789 (7)	157
O1*w*—H11⋯O5^ii^	0.84	2.00	2.723 (7)	144
O2*w*—H21⋯O3^iii^	0.84	2.05	2.762 (7)	142
O2*w*—H22⋯O6*w*	0.84	2.31	2.699 (7)	109
O3*w*—H31⋯O9*w*	0.84	1.89	2.710 (10)	165
O4*w*—H41⋯O4^ii^	0.84	2.03	2.693 (7)	136
O4*w*—H42⋯O4^iii^	0.84	2.02	2.713 (7)	139
O5*w*—H52⋯O7*w*	0.84	2.25	2.762 (9)	119
O6*w*—H61⋯O1^iv^	0.84	2.04	2.751 (7)	142
O6*w*—H62⋯O8*w*^ii^	0.84	2.09	2.825 (9)	146
O7*w*—H71⋯O3^iv^	0.84	1.99	2.818 (9)	169
O7*w*—H72⋯O8*w*^v^	0.84	2.36	3.169 (10)	162
O8*w*—H81⋯O5	0.84	2.22	2.778 (8)	124
O8*w*—H82⋯O9*w*	0.84	2.45	2.756 (11)	103
O9*w*—H91⋯O4*w*^vi^	0.84	2.29	2.895 (9)	130

Symmetry codes: (i) 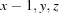; (ii) 
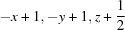
; (iii) 
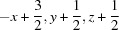
; (iv) 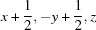; (v) 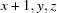; (vi) 
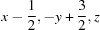
.

**Fig. 1.**
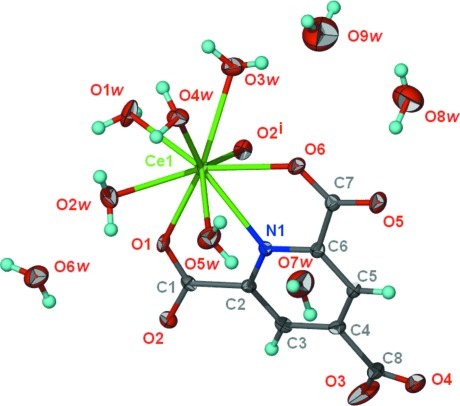
Thermal ellipsoid plot (Barbour, 2001) of the formular unit of polymeric Ce(H2O)5(C8H2NO6).4H2O at the 50% probability level; hydrogen atoms are drawn as spheres of arbitrary radius.

**Fig. 2.**
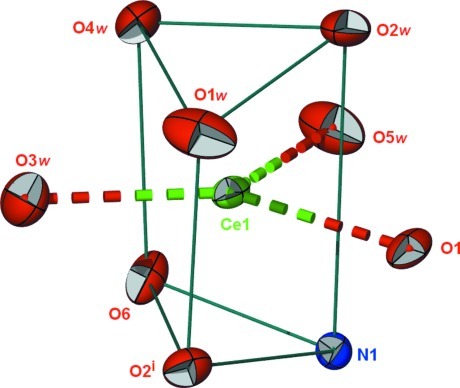
Tricapped trigonal prismatic geometry of the Ce(III) atom in Ce(H2O)5(C8H2NO6).4H2O.

## supplementary crystallographic information

### Comment

Some lanthanide coordination polymers of 2,4,6-pyridinetricarboxylic acid exhibit photoluminescence; the Sm, Eu, Tb and Ho derivatives are isostructural pentaaqua-coordinated tetrahydrates having the rare-earth atoms in tricapped trigonal prismatic geometries (Wang *et al.*, 2007). The cerium analog is also isostructural; The Ce(III) atom in Ce(H_2_O)_5_(C_8_H_2_NO_6_)^.^4H_2_O (Scheme I, Fig. 1) is *N*,*O*,*O*'-chelated by the carboxylate trianion and is coordinated by five water molecules. A carboxyl O atom from an adjacent trianion results in a chain running along the *a*-axis of the orthorhombic unit cell. The nine atoms surrounding the metal atom comprises a tricapped trigonal prismatic polyhedron (Fig. 2). The coordinated and lattice water molecules interact by themselves and with the carboxyl O-atoms by O–H···O hydrogen bonds to generate a three-dimensional network (Table 1).

### Experimental

Pyridinetricarboxylic acid was synthesized by using a reported method (Syper *et al.*, 1980). The compound (0.110 g, 0.5 mmol) was dissolved in water (7 ml) and added to a solution of cerium nitrate (0.054 g. 0.5 mmol) dissolved in 50% aqueous methanol (5 ml). The solution was heated for a hour, and then set aside for the growth of yellow prisms. These appeared after three weeks. mixture was refluxed for two hours. Yellow prisms like crystals were obtained after three weeks.

### Refinement

Carbon-bound H-atoms were placed in calculated positions (C—H 0.93 Å) and were included in the refinement in the riding model approximation, with *U*(H) set to 1.2*U*(C).

The water H-atoms were placed in chemically sensible positions on the basis of hydrogen bonds (O–H 0.8 Å); their temperature factors were fixed ats 1.5 times those of the parent O-atoms.

The anisotropic temperature factors of the O-atoms of the lattice water molecules were restrained to be nearly isotropic.

### Crystal data

**Table:**

[Ce(C_8_H_2_NO_6_)(H_2_O)_5_]·4H_2_O	*F*(000) = 1012
*M**_r_* = 510.37	*D*_x_ = 2.077 Mg m^−^^3^
Orthorhombic, *P**n**a*2_1_	Mo *K*α radiation, λ = 0.71073 Å
Hall symbol: P 2c -2n	Cell parameters from 7136 reflections
*a* = 6.8437 (3) Å	θ = 3.1–28.2°
*b* = 13.3207 (5) Å	µ = 2.87 mm^−^^1^
*c* = 17.9045 (7) Å	*T* = 293 K
*V* = 1632.23 (11) Å^3^	Prism, yellow
*Z* = 4	0.21 × 0.06 × 0.04 mm

### Data collection

**Table:**

Bruker APEXII diffractometer	3690 independent reflections
Radiation source: fine-focus sealed tube	3336 reflections with *I* > 2σ(*I*)
Graphite monochromator	*R*_int_ = 0.034
ω scans	θ_max_ = 27.5°, θ_min_ = 2.3°
Absorption correction: multi-scan (*SADABS*; Sheldrick, 1996)	*h* = −8→5
*T*_min_ = 0.584, *T*_max_ = 0.894	*k* = −17→16
19512 measured reflections	*l* = −23→22

### Refinement

**Table:**

Refinement on *F*^2^	Secondary atom site location: difference Fourier map
Least-squares matrix: full	Hydrogen site location: inferred from neighbouring sites
*R*[*F*^2^ > 2σ(*F*^2^)] = 0.033	H-atom parameters constrained
*wR*(*F*^2^) = 0.078	*w* = 1/[σ^2^(*F*_o_^2^) + (0.0295*P*)^2^ + 6.9973*P*] where *P* = (*F*_o_^2^ + 2*F*_c_^2^)/3
*S* = 1.07	(Δ/σ)_max_ = 0.001
3690 reflections	Δρ_max_ = 0.68 e Å^−^^3^
226 parameters	Δρ_min_ = −0.51 e Å^−^^3^
25 restraints	Absolute structure: Flack (1983), 1757 Friedel pairs
Primary atom site location: structure-invariant direct methods	Flack parameter: −0.05 (2)

### Fractional atomic coordinates and isotropic or equivalent isotropic displacement parameters (Å^2^)

**Table:**

	*x*	*y*	*z*	*U*_iso_*/*U*_eq_	
Ce1	0.48574 (3)	0.464973 (17)	0.49983 (4)	0.01583 (8)	
O1	0.5984 (6)	0.2872 (3)	0.5134 (2)	0.0230 (10)	
O2	0.6815 (6)	0.1363 (3)	0.4705 (2)	0.0245 (9)	
O3	0.5476 (11)	0.0845 (5)	0.1892 (4)	0.0538 (19)	
O4	0.6142 (9)	0.2190 (4)	0.1212 (3)	0.0446 (14)	
O5	0.4457 (8)	0.5584 (3)	0.2552 (3)	0.0346 (11)	
O6	0.4253 (7)	0.5458 (3)	0.3787 (2)	0.0279 (9)	
O1w	0.3428 (7)	0.4311 (4)	0.6266 (3)	0.0392 (12)	
H11	0.4322	0.4136	0.6560	0.059*	
H12	0.2597	0.3850	0.6233	0.059*	
O2w	0.7604 (7)	0.4543 (3)	0.5942 (3)	0.0336 (11)	
H21	0.7783	0.5110	0.6135	0.050*	
H22	0.8633	0.4356	0.5727	0.050*	
O3w	0.1808 (6)	0.5787 (4)	0.5174 (3)	0.0368 (13)	
H31	0.1733	0.6194	0.4817	0.055*	
H32	0.1907	0.6105	0.5577	0.055*	
O4w	0.5804 (7)	0.6370 (3)	0.5462 (3)	0.0308 (10)	
H41	0.5090	0.6528	0.5826	0.046*	
H42	0.6977	0.6360	0.5600	0.046*	
O5w	0.8185 (7)	0.5032 (4)	0.4421 (3)	0.0386 (11)	
H51	0.8272	0.5650	0.4335	0.058*	
H52	0.8305	0.4712	0.4019	0.058*	
O6w	1.0103 (8)	0.3123 (4)	0.6438 (3)	0.0423 (13)	
H61	1.0118	0.2610	0.6165	0.063*	
H62	0.9898	0.2961	0.6884	0.063*	
O7w	0.9175 (12)	0.5539 (6)	0.2974 (4)	0.0593 (19)	
H71	0.9540	0.5066	0.2697	0.089*	
H72	1.0053	0.5977	0.3007	0.089*	
O8w	0.2199 (9)	0.7304 (5)	0.2708 (4)	0.0668 (18)	
H81	0.3388	0.7149	0.2686	0.100*	
H82	0.1979	0.7848	0.2931	0.100*	
O9w	0.1128 (17)	0.7295 (6)	0.4192 (5)	0.113 (3)	
H91	0.1622	0.7812	0.4383	0.169*	
H92	−0.0087	0.7384	0.4195	0.169*	
N1	0.5422 (7)	0.3589 (3)	0.3789 (3)	0.0167 (9)	
C1	0.6225 (7)	0.2232 (4)	0.4618 (3)	0.0174 (11)	
C2	0.5789 (8)	0.2597 (4)	0.3837 (3)	0.0159 (10)	
C3	0.5835 (8)	0.1987 (4)	0.3213 (4)	0.0205 (11)	
H3	0.6024	0.1298	0.3262	0.025*	
C4	0.5595 (8)	0.2415 (4)	0.2508 (3)	0.0201 (11)	
C5	0.5308 (8)	0.3449 (4)	0.2457 (3)	0.0192 (11)	
H5	0.5228	0.3763	0.1994	0.023*	
C6	0.5142 (7)	0.4002 (4)	0.3112 (3)	0.0172 (10)	
C7	0.4583 (8)	0.5113 (4)	0.3140 (3)	0.0199 (11)	
C8	0.5716 (9)	0.1763 (5)	0.1811 (4)	0.0272 (13)	

### Atomic displacement parameters (Å^2^)

**Table:**

	*U*^11^	*U*^22^	*U*^33^	*U*^12^	*U*^13^	*U*^23^
Ce1	0.02002 (12)	0.01382 (12)	0.01366 (12)	−0.00054 (9)	−0.0016 (3)	−0.0023 (2)
O1	0.041 (2)	0.0170 (17)	0.011 (3)	0.0042 (15)	−0.0039 (16)	−0.0025 (15)
O2	0.029 (2)	0.019 (2)	0.025 (2)	0.0062 (16)	−0.0011 (17)	0.0015 (16)
O3	0.082 (5)	0.035 (3)	0.045 (4)	−0.009 (3)	0.024 (3)	−0.021 (3)
O4	0.067 (4)	0.043 (3)	0.024 (3)	0.027 (3)	0.005 (2)	0.000 (2)
O5	0.058 (3)	0.027 (2)	0.019 (2)	0.011 (2)	0.003 (2)	0.0068 (19)
O6	0.043 (2)	0.021 (2)	0.019 (2)	0.0103 (19)	0.0019 (19)	−0.0015 (17)
O1w	0.040 (3)	0.062 (3)	0.016 (2)	−0.012 (2)	0.0009 (19)	0.000 (2)
O2w	0.038 (3)	0.030 (2)	0.033 (3)	0.003 (2)	−0.019 (2)	−0.010 (2)
O3w	0.033 (2)	0.040 (3)	0.038 (4)	0.0065 (19)	0.0004 (19)	−0.012 (2)
O4w	0.032 (2)	0.025 (2)	0.036 (3)	0.0027 (18)	−0.004 (2)	−0.0116 (19)
O5w	0.031 (2)	0.051 (3)	0.033 (3)	−0.006 (2)	0.005 (2)	−0.007 (2)
O6w	0.055 (3)	0.034 (3)	0.038 (3)	0.004 (2)	0.003 (2)	−0.007 (2)
O7w	0.065 (4)	0.065 (4)	0.048 (4)	0.003 (3)	0.010 (3)	0.003 (3)
O8w	0.057 (4)	0.067 (4)	0.077 (4)	0.014 (3)	−0.002 (3)	0.001 (3)
O9w	0.172 (8)	0.076 (5)	0.090 (5)	0.046 (5)	−0.008 (6)	−0.014 (4)
N1	0.018 (2)	0.016 (2)	0.017 (2)	0.0018 (17)	−0.0022 (17)	−0.0001 (18)
C1	0.015 (2)	0.014 (3)	0.023 (3)	−0.0001 (19)	−0.002 (2)	0.002 (2)
C2	0.017 (2)	0.013 (2)	0.018 (3)	0.0021 (19)	0.000 (2)	−0.002 (2)
C3	0.018 (3)	0.018 (3)	0.026 (3)	0.001 (2)	−0.001 (2)	0.001 (2)
C4	0.018 (2)	0.022 (3)	0.020 (3)	0.003 (2)	0.004 (2)	−0.002 (2)
C5	0.021 (3)	0.022 (3)	0.015 (3)	0.003 (2)	0.000 (2)	−0.001 (2)
C6	0.018 (2)	0.019 (3)	0.015 (3)	−0.0012 (19)	0.001 (2)	0.001 (2)
C7	0.024 (3)	0.018 (3)	0.018 (3)	0.001 (2)	0.001 (2)	0.003 (2)
C8	0.028 (3)	0.034 (3)	0.020 (3)	0.006 (3)	−0.004 (2)	−0.010 (3)

### Geometric parameters (Å, º)

**Table:**

Ce1—O6	2.456 (4)	O4w—H42	0.8399
Ce1—O1	2.502 (4)	O5w—H51	0.8400
Ce1—O1w	2.512 (5)	O5w—H52	0.8399
Ce1—O4w	2.523 (4)	O6w—H61	0.8399
Ce1—O2w	2.532 (4)	O6w—H62	0.8401
Ce1—O2^i^	2.536 (4)	O7w—H71	0.8399
Ce1—O5w	2.552 (5)	O7w—H72	0.8401
Ce1—O3w	2.598 (4)	O8w—H81	0.8400
Ce1—N1	2.615 (5)	O8w—H82	0.8400
O1—C1	1.268 (6)	O9w—H91	0.8400
O2—C1	1.237 (6)	O9w—H92	0.8399
O2—Ce1^ii^	2.536 (4)	N1—C6	1.344 (7)
O3—C8	1.242 (9)	N1—C2	1.347 (7)
O4—C8	1.249 (8)	C1—C2	1.509 (8)
O5—C7	1.228 (7)	C2—C3	1.383 (8)
O6—C7	1.266 (7)	C3—C4	1.394 (8)
O1w—H11	0.8399	C3—H3	0.9300
O1w—H12	0.8400	C4—C5	1.395 (8)
O2w—H21	0.8399	C4—C8	1.523 (8)
O2w—H22	0.8400	C5—C6	1.390 (8)
O3w—H31	0.8401	C5—H5	0.9300
O3w—H32	0.8399	C6—C7	1.530 (8)
O4w—H41	0.8401		
			
O6—Ce1—O1	123.53 (13)	Ce1—O2w—H22	109.5
O6—Ce1—O1w	144.17 (17)	H21—O2w—H22	109.5
O1—Ce1—O1w	82.09 (16)	Ce1—O3w—H31	109.5
O6—Ce1—O4w	86.32 (15)	Ce1—O3w—H32	109.5
O1—Ce1—O4w	138.49 (14)	H31—O3w—H32	109.5
O1w—Ce1—O4w	88.02 (17)	Ce1—O4w—H41	109.4
O6—Ce1—O2w	137.65 (17)	Ce1—O4w—H42	109.1
O1—Ce1—O2w	69.70 (14)	H41—O4w—H42	109.5
O1w—Ce1—O2w	71.10 (17)	Ce1—O5w—H51	109.4
O4w—Ce1—O2w	68.94 (14)	Ce1—O5w—H52	109.5
O6—Ce1—O2^i^	84.95 (15)	H51—O5w—H52	109.5
O1—Ce1—O2^i^	76.68 (13)	H61—O6w—H62	110.3
O1w—Ce1—O2^i^	76.83 (15)	H71—O7w—H72	110.5
O4w—Ce1—O2^i^	139.67 (14)	H81—O8w—H82	114.1
O2w—Ce1—O2^i^	135.89 (16)	H91—O9w—H92	106.3
O6—Ce1—O5w	72.86 (17)	C6—N1—C2	119.1 (5)
O1—Ce1—O5w	87.32 (17)	C6—N1—Ce1	120.3 (4)
O1w—Ce1—O5w	138.51 (17)	C2—N1—Ce1	120.3 (3)
O4w—Ce1—O5w	73.92 (17)	O2—C1—O1	125.5 (5)
O2w—Ce1—O5w	67.60 (18)	O2—C1—C2	118.9 (5)
O2^i^—Ce1—O5w	139.01 (16)	O1—C1—C2	115.6 (4)
O6—Ce1—O3w	73.51 (15)	N1—C2—C3	121.9 (5)
O1—Ce1—O3w	141.96 (15)	N1—C2—C1	114.4 (4)
O1w—Ce1—O3w	71.48 (17)	C3—C2—C1	123.7 (5)
O4w—Ce1—O3w	68.68 (14)	C2—C3—C4	119.2 (5)
O2w—Ce1—O3w	123.26 (15)	C2—C3—H3	120.4
O2^i^—Ce1—O3w	71.08 (13)	C4—C3—H3	120.4
O5w—Ce1—O3w	130.50 (17)	C3—C4—C5	118.7 (5)
O6—Ce1—N1	62.00 (14)	C3—C4—C8	120.1 (5)
O1—Ce1—N1	61.53 (13)	C5—C4—C8	121.1 (5)
O1w—Ce1—N1	135.14 (16)	C6—C5—C4	118.6 (5)
O4w—Ce1—N1	136.56 (16)	C6—C5—H5	120.7
O2w—Ce1—N1	114.37 (15)	C4—C5—H5	120.7
O2^i^—Ce1—N1	70.25 (14)	N1—C6—C5	122.2 (5)
O5w—Ce1—N1	68.94 (16)	N1—C6—C7	113.8 (5)
O3w—Ce1—N1	122.29 (14)	C5—C6—C7	124.1 (5)
C1—O1—Ce1	127.3 (3)	O5—C7—O6	125.9 (5)
C1—O2—Ce1^ii^	142.4 (4)	O5—C7—C6	118.9 (5)
C7—O6—Ce1	128.2 (4)	O6—C7—C6	115.1 (5)
Ce1—O1w—H11	109.4	O3—C8—O4	125.4 (7)
Ce1—O1w—H12	109.4	O3—C8—C4	117.3 (6)
H11—O1w—H12	109.5	O4—C8—C4	117.2 (6)
Ce1—O2w—H21	109.4		
			
O6—Ce1—O1—C1	−4.4 (5)	Ce1^ii^—O2—C1—C2	105.5 (6)
O1w—Ce1—O1—C1	148.5 (5)	Ce1—O1—C1—O2	178.7 (4)
O4w—Ce1—O1—C1	−133.6 (4)	Ce1—O1—C1—C2	0.4 (7)
O2w—Ce1—O1—C1	−138.8 (5)	C6—N1—C2—C3	−1.8 (8)
O2^i^—Ce1—O1—C1	70.2 (4)	Ce1—N1—C2—C3	171.2 (4)
O5w—Ce1—O1—C1	−71.7 (5)	C6—N1—C2—C1	175.7 (5)
O3w—Ce1—O1—C1	102.7 (5)	Ce1—N1—C2—C1	−11.3 (6)
N1—Ce1—O1—C1	−4.3 (4)	O2—C1—C2—N1	−171.1 (5)
O1—Ce1—O6—C7	−6.2 (6)	O1—C1—C2—N1	7.3 (7)
O1w—Ce1—O6—C7	−135.7 (5)	O2—C1—C2—C3	6.3 (8)
O4w—Ce1—O6—C7	142.8 (5)	O1—C1—C2—C3	−175.2 (5)
O2w—Ce1—O6—C7	90.0 (5)	N1—C2—C3—C4	3.4 (8)
O2^i^—Ce1—O6—C7	−76.6 (5)	C1—C2—C3—C4	−173.9 (5)
O5w—Ce1—O6—C7	68.5 (5)	C2—C3—C4—C5	−0.3 (8)
O3w—Ce1—O6—C7	−148.3 (5)	C2—C3—C4—C8	177.7 (5)
N1—Ce1—O6—C7	−6.3 (5)	C3—C4—C5—C6	−4.2 (8)
O6—Ce1—N1—C6	1.1 (4)	C8—C4—C5—C6	177.8 (5)
O1—Ce1—N1—C6	−178.8 (4)	C2—N1—C6—C5	−3.0 (8)
O1w—Ce1—N1—C6	141.2 (4)	Ce1—N1—C6—C5	−176.0 (4)
O4w—Ce1—N1—C6	−47.1 (5)	C2—N1—C6—C7	175.5 (5)
O2w—Ce1—N1—C6	−131.6 (4)	Ce1—N1—C6—C7	2.6 (6)
O2^i^—Ce1—N1—C6	96.0 (4)	C4—C5—C6—N1	6.0 (8)
O5w—Ce1—N1—C6	−80.1 (4)	C4—C5—C6—C7	−172.4 (5)
O3w—Ce1—N1—C6	45.3 (4)	Ce1—O6—C7—O5	−171.5 (5)
O6—Ce1—N1—C2	−171.8 (5)	Ce1—O6—C7—C6	9.8 (7)
O1—Ce1—N1—C2	8.3 (4)	N1—C6—C7—O5	173.9 (5)
O1w—Ce1—N1—C2	−31.7 (5)	C5—C6—C7—O5	−7.6 (8)
O4w—Ce1—N1—C2	140.1 (4)	N1—C6—C7—O6	−7.4 (7)
O2w—Ce1—N1—C2	55.5 (4)	C5—C6—C7—O6	171.1 (5)
O2^i^—Ce1—N1—C2	−76.9 (4)	C3—C4—C8—O3	19.8 (9)
O5w—Ce1—N1—C2	107.0 (4)	C5—C4—C8—O3	−162.2 (7)
O3w—Ce1—N1—C2	−127.5 (4)	C3—C4—C8—O4	−156.7 (6)
Ce1^ii^—O2—C1—O1	−72.8 (9)	C5—C4—C8—O4	21.2 (9)

Symmetry codes: (i) *x*−1/2, −*y*+1/2, *z*; (ii) *x*+1/2, −*y*+1/2, *z*.

### Hydrogen-bond geometry (Å, º)

**Table:**

*D*—H···*A*	*D*—H	H···*A*	*D*···*A*	*D*—H···*A*
O1*w*—H12···O6*w*^iii^	0.84	2.00	2.789 (7)	157
O1*w*—H11···O5^iv^	0.84	2.00	2.723 (7)	144
O2*w*—H21···O3^v^	0.84	2.05	2.762 (7)	142
O2*w*—H22···O6*w*	0.84	2.31	2.699 (7)	109
O3*w*—H31···O9*w*	0.84	1.89	2.710 (10)	165
O4*w*—H41···O4^iv^	0.84	2.03	2.693 (7)	136
O4*w*—H42···O4^v^	0.84	2.02	2.713 (7)	139
O5*w*—H52···O7*w*	0.84	2.25	2.762 (9)	119
O6*w*—H61···O1^ii^	0.84	2.04	2.751 (7)	142
O6*w*—H62···O8*w*^iv^	0.84	2.09	2.825 (9)	146
O7*w*—H71···O3^ii^	0.84	1.99	2.818 (9)	169
O7*w*—H72···O8*w*^vi^	0.84	2.36	3.169 (10)	162
O8*w*—H81···O5	0.84	2.22	2.778 (8)	124
O8*w*—H82···O9*w*	0.84	2.45	2.756 (11)	103
O9*w*—H91···O4*w*^vii^	0.84	2.29	2.895 (9)	130

Symmetry codes: (ii) *x*+1/2, −*y*+1/2, *z*; (iii) *x*−1, *y*, *z*; (iv) −*x*+1, −*y*+1, *z*+1/2; (v) −*x*+3/2, *y*+1/2, *z*+1/2; (vi) *x*+1, *y*, *z*; (vii) *x*−1/2, −*y*+3/2, *z*.
